# Study of the Metabolomics of Equine Preovulatory Follicular Fluid: A Way to Improve Current In Vitro Maturation Media

**DOI:** 10.3390/ani10050883

**Published:** 2020-05-19

**Authors:** Pablo Fernández-Hernández, María Jesús Sánchez-Calabuig, Luis Jesús García-Marín, María J. Bragado, Alfonso Gutiérrez-Adán, Óscar Millet, Chiara Bruzzone, Lauro González-Fernández, Beatriz Macías-García

**Affiliations:** 1 Research Group of Intracellular Signaling and Technology of Reproduction, Research Institute INBIO G + C, University of Extremadura, 10003 Cáceres, Spain; pablofh@unex.es (P.F.-H.); ljgarcia@unex.es (L.J.G.-M.); jbragado@unex.es (M.J.B.); lgonfer@unex.es (L.G.-F.); 2 Department of Animal Medicine, Faculty of Veterinary Sciences, University of Extremadura, 10003 Cáceres, Spain; 3 Department of Animal Reproduction, INIA, 28040 Madrid, Spain; mariasanchezcalabuig@gmail.com (M.J.S.-C.); agutierr@inia.es (A.G.-A.); 4 Department of Animal Medicine and Surgery, Faculty of Veterinary Sciences, University Complutense of Madrid, 28040 Madrid, Spain; 5 Department of Physiology, Faculty of Veterinary Sciences, University of Extremadura, 10003 Cáceres, Spain; 6 Department of Biochemistry and Molecular Biology and Genetics, Faculty of Veterinary Sciences, University of Extremadura, 10003 Cáceres, Spain; 7 Precision Medicine and Metabolism Lab., CICbioGUNE, 48160 Vizcaya, Spain; omillet@cicbiogune.es (Ó.M.); cbruzzone@cicbiogune.es (C.B.)

**Keywords:** IVM, oocytes, equine, metabolomic

## Abstract

**Simple Summary:**

Commercial in vitro embryo production in horses by ICSI (intracytoplasmic sperm injection) is currently used to produce embryos clinically. However, the successful pregnancy and foaling rates obtained after ICSI are only 10% of the oocytes matured in vitro. Conditions used for oocyte in vitro maturation are not optimized for equine oocytes. Hence, in the present work, we aimed to elucidate the major metabolites present in equine preovulatory follicular fluid obtained from postmortem mares using proton nuclear magnetic resonance spectroscopy (^1^H-NMR). Twenty-two metabolites were identified; among these, nine of them are not included in the composition of in vitro maturation media. Hence, our data suggest that the currently used media for equine oocyte maturation can be further improved.

**Abstract:**

Production of equine embryos in vitro is currently a commercial technique and a reliable way of obtaining offspring. In order to produce those embryos, immature oocytes are retrieved from postmortem ovaries or live mares by ovum pick-up (OPU), matured in vitro (IVM), fertilized by intracytoplasmic sperm injection (ICSI), and cultured until day 8–10 of development. However, at best, roughly 10% of the oocytes matured in vitro and followed by ICSI end up in successful pregnancy and foaling, and this could be due to suboptimal IVM conditions. Hence, in the present work, we aimed to elucidate the major metabolites present in equine preovulatory follicular fluid (FF) obtained from postmortem mares using proton nuclear magnetic resonance spectroscopy (^1^H-NMR). The results were contrasted against the composition of the most commonly used media for equine oocyte IVM: tissue culture medium 199 (TCM-199) and Dulbecco’s modified eagle medium/nutrient mixture F-12 Ham (DMEM/F-12). Twenty-two metabolites were identified in equine FF; among these, nine of them are not included in the composition of DMEM/F-12 or TCM-199 media, including (mean ± SEM): acetylcarnitine (0.37 ± 0.2 mM), carnitine (0.09 ± 0.01 mM), citrate (0.4 ± 0.04 mM), creatine (0.36 ± 0.14 mM), creatine phosphate (0.36 ± 0.05 mM), fumarate (0.05 ± 0.007 mM), glucose-1-phosphate (6.9 ± 0.4 mM), histamine (0.25 ± 0.01 mM), or lactate (27.3 ± 2.2 mM). Besides, the mean concentration of core metabolites such as glucose varied (4.3 mM in FF vs. 5.55 mM in TCM-199 vs. 17.5 mM in DMEM/F-12). Hence, our data suggest that the currently used media for equine oocyte IVM can be further improved.

## 1. Introduction

Production of equine embryos in vitro is currently a commercial technique and a reliable way of producing embryos for vitrification or uterine/oviductal transfer [1]. In order to produce those embryos, immature oocytes are retrieved from postmortem ovaries or live mares by ovum pick-up (OPU) [2], matured in vitro (IVM), fertilized by intracytoplasmic sperm injection (ICSI), and cultured until day 8–10 of development [3]. However, among all the oocytes used for ICSI, in the best of the scenarios, roughly 10% of them end up in successful pregnancy and foaling [1,4]. Surprisingly, when in vivo matured equine oocytes are transferred to oviducts of live mares to produce equine offspring, the likelihood of pregnancy rises to 75%, contrasting with the 40% obtained when the oocytes transferred are matured in vitro [1]. These results highlight the fact that the media and conditions used for oocytes matured in vitro (IVM) largely differ from the physiological conditions required for correct nuclear and cytoplasmic maturation in horses, therefore decreasing the oocyte’s developmental competence.

It has to be noted that the base media more commonly used for equine IVM are tissue culture medium 199 (TCM-199) or Dulbecco’s modified eagle medium/nutrient mixture F-12 Ham (DMEM/F-12), which are generally chosen depending on the preferences of the laboratory where IVM is performed, and core differences exist among them [5]. Furthermore, none of these media have been developed specifically for equine IVM; instead, they were developed for cell culture, albeit equine cumulus–oocyte complexes (COCs) are capable of maturing with similar efficiency in either media [3,6,7].

To try to better understand the physiological conditions that equine COCs require and improve current IVM conditions, several reports have tried to address the metabolic requirements of equine COCs in vitro [5,6,8], the differences between the proteomic profiles of equine COCs maturated in vivo or in vitro [8], or the differential expression and localization of glycosidic residues in equine COCs matured in vitro vs. in vivo [9], among other approaches. All these reports have revealed a specific metabolic profile of equine COCs matured in vitro and important differences between equine COCs matured in vitro vs. in vivo. However, no research has been conducted to develop a defined oocyte equine-specific maturation medium. Hence, in the present work, we aimed to elucidate the metabolomic composition of equine preovulatory follicular fluid (FF). To do this, the major metabolites present in equine preovulatory follicular fluid were analyzed by high-field proton nuclear magnetic resonance spectroscopy (^1^H-NMR) and the results were contrasted against the composition of the formerly mentioned media according to the manufacturer’s specifications.

## 2. Materials and Methods

### 2.1. Collection of Equine Follicular Fluid

Follicular fluid was obtained immediately postmortem at a commercial slaughterhouse, on four separate days. At the time of evisceration, the entire mare reproductive tract was extracted and carefully inspected. The ovaries were examined and those tracts having a preovulatory follicle ≥35 mm in diameter, associated with uterine edema on examination of the opened endometrial surface (vivid endometrial folds with a gelatinous appearance), were sampled as preovulatory. The fluid was collected using a 10 mL plastic syringe attached to a 20 g hypodermic needle. The fluid obtained was separated into 1.5 mL Eppendorf tubes and centrifuged for 2 min in a microcentrifuge at room temperature (RT) to remove large cellular masses. The supernatant was retrieved, transferred to a clean tube, and placed in dry ice until its arrival at the laboratory (4–5 h). Once at the laboratory, the fluid was thawed and centrifuged at 16,000× *g* at 4 °C for 20 min, and the supernatant was transferred to a clean tube. The samples were then kept at −80 °C until analysis.

### 2.2. Sample Preparation

Samples of follicular fluid from six different mares (one sample per mare) at the preovulatory stage (PRE) were used (*n* = 6). For the preparation of the nuclear magnetic resonance samples (NMR), the follicular fluids were pretreated. A methanol extraction was performed with the following protocol: samples were defrosted at room temperature for 30 min slowly on ice and 170 µL of follicular fluid of each sample were placed in a 1.5 mL Eppendorf tube and 1.3 mL of a mixture of methanol:deuterated water in a ratio 2:1 was added to the follicular fluid. The Eppendorf tube with the extraction was placed at 4 °C with agitation for 4 h. The mixture was centrifuged at 4 °C at 25,000× *g* for 30 min. The supernatant was transferred to a new 2 mL Eppendorf tube and the samples were plunged in liquid N_2_. Once the mixtures were frozen, samples were subjected to lyophilization. For sample analysis, the lyophilized product was resuspended with 500 μL of 0.2 M potassium phosphate buffer in deuterium oxide (D_2_O) with a pH of 7.4 ± 0.5 and 1.11 μL of TSP (3-(Trimethylsilyl) propanoic acid), to reach a final volume of 500 μL. Samples were briefly vortexed and 500 μL of the follicular fluid/buffer mixture were finally pipetted into a 5 mm NMR tube. In all cases, sample preparation was manually done at RT.

### 2.3. NMR Measurements

Samples were measured at 298 K in an 800 MHz Bruker spectrometer (AVANCE III, Bruker Biospin GmbH, Reinsthetten, Germany) equipped with a ^1^H detected cryoprobe with z-gradient and automatic tuning and matching unit. Optimization of experimental conditions included automated tuning and matching, automated locking, and automated shimming using TopShim. The 90° hard pulse was optimized to be sample-specific and the presaturation field strength was adjusted to 25 Hz. To minimize the interference of the water content in the NMR spectrum, solvent suppression techniques were applied.

For each sample, one-dimensional (1D) ^1^H-NMR spectra were collected using a Carr–Purcell–Meiboom–Gill (CPMG) pulse sequence; 2D *J*-resolved included 800 × 40 points. Data analysis was done using the TopSpin 3.5 software (Bruker Biospin GmbH, Reinsthetten, Germany). Free induction decays were multiplied by an exponential function equivalent to 0.3 Hz line-broadening before applying a Fourier transformation. All transformed spectra were automatically corrected for phase and baseline distortions and referenced to the DSS singlet at 0 ppm for further analysis.

The 2D-*J*res experiment was also routinely included in the acquisition package, along with the 1D ^1^H-NOESY. This experiment separates J-couplings and chemical shifts in the 2D plane and provides a useful and simplified proton-decoupled projection spectrum. A standard pulse sequence with a water peak suppression was used. After 16 dummy scans, 2 free induction decays (FIDs) were accumulated into 8 k × 40 data points at a spectral width of 16 ppm.

For assignment purposes, a battery of experiments including 2D-^1^H, ^13^C-HSQC, 2D-^1^H, ^1^H-TOCSY, and 2D ^1^H-^1^H-NOESY were recorded in a Bruker Avance III 800 MHz spectrometer (Figure S1). The chemical shift, multiplet type, and number of contributing nuclei to each metabolite are provided in Table 1 and were determined following previously validated methods [10,11,12].

### 2.4. Commercial Media Composition

The composition of commercial media routinely used in our laboratory: TCM-199 with Earle´s salts (Ref. 31150022; Thermo Fisher Scientific (Waltham, MA, USA)) and DMEM/F-12 (Ref. 11320033; Thermo Fisher Scientific (Waltham, MA, USA)) were directly extracted from the manufacturer´s website.

### 2.5. Statistical Analysis

Data were analyzed using descriptive statistics to establish the mean, standard error of the mean, minimum, and maximum for each metabolite using the software Sigma Plot (ver. 12.0) for windows (Systat Software, Chicago, IL, USA).

## 3. Results

### 3.1. Metabolite Identification

The chemical shift assignment for each metabolite was performed using a random follicular fluid sample; the spectra of the FF (Figure S1) was contrasted against the identified metabolites that were chosen based on our previous study in horses [13]. The list of the chemical shift for each proton nucleus of each metabolite is provided in Table 1. The measured concentrations of 22 metabolites, which were selected based on the report of González-Fernández et al. (2020) [13], and the known identification capabilities of the NMR facility for preovulatory follicular fluid samples are presented in Table 2. Pyruvate and succinate are characterized by one single peak with the same chemical shift and cannot be discriminated; therefore, they are presented as the sum of both metabolites. All metabolites were detected in all the samples except for acetylcarnitine, which could not be detected in one sample submitted to NMR analysis.

### 3.2. Comparison of the Metabolites Present in Commercial Media and Equine Preovulatory Follicular Fluid

Among the 22 metabolites identified in native preovulatory FF, nine of them are not present in TCM-199 or DMEM/F-12 according to the manufacturer’s specifications (Table 3). These metabolites were: acetylcarnitine, carnitine, citrate, creatine, creatine phosphate, fumarate, glucose-1-phosphate, histamine, and lactate. Other metabolites such as acetate is present in FF and TCM-199 but not in DMEM/F-12, while pyruvate is included in the composition of DMEM/F-12 and possibly is present in FF (as it cannot be discriminated from succinate) but not in TCM-199. Vivid differences exist in the concentration of core metabolites such as lactate (27.3 mM in FF vs. 0 mM in TCM-199 and DMEM/F-12), glucose (4.3 mM in FF vs. 5.55 mM in TCM-199 vs. 17.5 mM in DMEM/F-12), alanine (1.1 mM in FF vs. 0.28 mM in TCM-199 vs. 0.05 mM in DMEM/F-12), aspartate (2.7 mM in FF vs. 0.22 mM in TCM-199 vs. 0.05 mM in DMEM/F-12), or glycine (3.2 mM in FF vs. 0.67 mM in TCM-199 vs. 0.25 mM in DMEM/F-12).

## 4. Discussion

In the present work, the metabolome of equine preovulatory FF was investigated using ^1^H-NMR. Our work revealed the presence of at least 22 metabolites including carbohydrates, amino acids, and intermediate metabolites (Table 2). The metabolome of equine FF at different dominant follicular stages (early dominant, late dominant, and healthy preovulatory stage) has previously been described by Gérard et al. in 2002 [14]. In their work, they did not detect apparent differences in the pattern or concentration of the metabolites detected among the studied stages. These authors described eight peaks corresponding to chemical groups of sugar chains and N-acetyl groups of glycoconjugates, CH3 groups of lipoproteins, trimethylamines, acetate, alanine, creatine/creatinine, and polyamines, plus a non-identified peak at 3.1 ppm, but quantitative identification of the peaks is not provided [14]. In our work, we detected 21 peaks (as succinate and pyruvate were overlapped; Table 1); an explanation for the differences found in the number of peaks (8 vs. 21) can be easily explained as Gérard et al. (2002) used a 200 MHz Bruker spectrometer (in our work we used an 800 MHz spectrometer and a cryoprobe) and the samples were directly diluted in deuterated water (instead of being previously subjected to a methanol extraction and lyophilization as in the present work), likely resulting in higher water interferences and lower spectra resolution [14]. Hence, in the formerly mentioned work, some peaks as citrate are suspected, while in our work, it was detected in all samples due to a better resolution of current NMR spectrometers (Table 1 and Table 2). It must be mentioned that citrate and fumarate are not routinely added to base equine IVM media (Table 3) as both are intermediate metabolites produced by the metabolism of glucose in the tricarboxylic acid cycle (TCA); specifically, citrate comes from acetyl-CoA and oxaloacetate in the tricarboxylic acid cycle (TCA). Citrate acts as a key substrate for epigenetic modifiers in the oocyte [15,16] and is a link between TCA, β-hydroxybutyrate, and lipid metabolism in FF [17,18], so adequate supplementation could be important during equine IVM and should be added to TCM-199 (Table 3). Regarding pyruvate, this molecule has been previously reported to range between 0.03 and 0.13 mM in FF from early and late dominant equine follicles, respectively [19]. This metabolite has been demonstrated to be crucial for adequate oocyte metabolism [5] and is also involved in active reactive oxygen species scavenging [20]. In equine oocytes, it has been demonstrated that when DMEM/F-12 is supplemented with pyruvate at 0.15 mM, this induces an increase in glycolytic activity, without affecting mitochondrial oxidative phosphorylation [5]. The concentration of pyruvate above reported for equine FF [19] coincides with our work in which 0.16 mM ± 0.03 was observed. However, in our experiments, succinate could not be discriminated from pyruvate, and thus, exact values cannot be provided; nevertheless, as per previous reports, pyruvate addition to equine IVM media and the concentration at which it is supplemented needs to be seriously considered.

The research group of Gérard et al. also published in 2015 another report in which they performed a comparative metabolomic study of porcine, equine, and bovine FF [21]. In this report, even when the extraction method remained the same as in their previous work, a 500 mHz ^1^H-NMR spectrometer was used and the resolution of the analysis was improved. In this work, they described the presence and concentration of some metabolites that coincide with the ones reported in the present work such as acetate, alanine, citrate, and glucose (alpha and beta, while in our work, glucose and glucose-1-phosphate were detected) [21]. However, the concentration of other metabolites reported in their work such as histidine (0.05 ± 0.009 mM in our work vs. 0.27 mM [21]) and valine (0.13 ± 0.02 mM in our work vs. 0.37 mM [21]) do not agree with our findings. These differences can be attributed to the different equipment used, the sample extraction method, or the fact that Gérard et al. (2015) recovered the FF by transvaginal aspiration of follicles around 33 mm and in our work postmortem FF was obtained from bigger follicles (35–45 mm). Interestingly, lactate concentration largely differs in our report compared with that obtained by Gerard et al. [21] (27.3 mM ± 2.2 vs. 0.70 mM ± 0.18, respectively), the time between mare decease and sample obtention being around 30 min. This time lapse could lead to lactate postmortem accumulation [22] as previously observed for equine oviductal fluid [13]. Nevertheless, the high lactate concentration observed in the present work (19.3–35.02 mM) cannot be solely explained as the metabolism of the typical amount of available glucose (2 molecules of lactate per 1 of glucose consumed [17]), as the concentration of glucose observed in our work matches previous reports [14,19,21,23], and thus appears to reflect a high level of lactate in the equine FF. However, it has to be noticed that the equine FF analyzed by Gérard et al. (2015) [21] was retrieved when the dominant follicle reached 33 mm, while it has been demonstrated that in equine follicles reaching 39 mm 24 h after equine crude gonadotropin administration, the lactate values reach 4 mM, this concentration of lactate being closely related to adequate meiosis resumption in equine oocytes [19]. Similar findings among lactate production during IVM and oocyte competence in the horse have been recently reported in vitro [5], highlighting the important relationship existing between anaerobic glycolysis and oocyte meiotic competency in the horse, strongly suggesting that FF may be providing energy to the oocyte in the form of lactate as postulated in humans [17]. Furthermore, high lactate concentrations in the FF have been linked to improved pregnancy rates in humans [24], and this metabolite could be an important additive contributing to osmolarity adjustment, as reported in human oviductal fluid [25]; hence, lactate would need to be supplemented to equine IVM media (Table 3).

Notably in our NMR spectra, different intracellular metabolites such as glucose-1-phosphate, creatine, creatine phosphate, and carnitine were found. These metabolites cannot be incorporated into the oocytes in their molecular form and may have been released from the granulosa cells due to cellular damage. However, recent investigations have demonstrated that a wide variety of intracellular metabolites are also present in the oviductal fluid of cows and horses [13,26], embedded in extracellular vesicles [27]. The compounds included in these vesicles could enter the oocytes/embryos via fusion as previously demonstrated in mouse embryos [28], contributing to the oocyte’s developmental competence and metabolism as observed in cows [29]. It is known that carnitine plays a major role in the catabolism of lipids, allowing the transport of fatty acids from the cytosol to the mitochondria, where they are metabolized through beta-oxidation, which has also been identified in human FF [30]. Surprisingly, this work demonstrated the presence of carnitine in the FF but did not find expression of the enzymes involved in the carnitine synthesis either in oocytes or in cumulus cells. In contrast, the enzymes related to beta-oxidation were highly expressed in the oocyte and cumulus cells as also demonstrated in horses [8]. Therefore, this compound may also need to be somehow included in equine IVM media to ensure adequate oocyte lipid metabolism [30], as low concentration of carnitine in the FF has been associated with low reproductive performance outcome in sows [31]. The presence of creatine in the equine FF has been previously reported at similar concentrations to the ones reported in the present work [14]. Creatine and creatine phosphate are produced as a result of arginine and glycine metabolism. Even when arginine was not found in equine FF (Table 1 and Table 2), arginine was present in TCM-199 (0.33 mM) and DMEM/F-12 (0.7 mM). Interestingly, arginine depletion during the final 6 h of IVM of human oocytes was associated with a higher maturation potential [32,33]. Hence, even when arginine was not detected in our experiments (Table 1), our data suggest that an active metabolism of arginine could be occurring during in vivo maturation of equine oocytes explaining the depletion of this amino acid in the FF and thus, the arginine present in the equine IVM media (TCM-199 and DMEM/F-12) could be needed. Glycine is known to be an organic osmolyte that regulates osmolarity in cells and embryos [34] and is one of the most abundant amino acids in follicular and uterine fluids [35]. Interestingly, its concentration in the FF has been demonstrated to predict the cleavage rate of oocytes after insemination (being a stronger marker of cleavage capacity in lower grade oocytes) as well as to be a good marker of the blastocyst rate in bovine [36]. Hence, considering the vivid difference existing in glycine concentration among TCM-199, DMEM/F-12, and native equine preovulatory FF (Table 3), the concentration at which this amino acid is added to equine IVM media should be carefully evaluated.

Interestingly, glucose-1-phosphate was detected in equine preovulatory FF (Table 1). This molecule is derived from glycogen, which is generally metabolized in the liver but is also a metabolic source used by granulosa cells in humans, pigs, bulls, and mice [15,37,38]. Thus, in view of our data, the equine oocyte also relies on glycogen metabolism as is also proposed in previous reports [5]. This is an interesting finding as, in our experiments, the amount of glucose-1-phosphate found (6.9 mM ± 0.4) surpassed the quantity of glucose (4.3 ± 0.4 mM), indicating that glycogen metabolism could support equine oocyte maturation in vivo, and more research is needed in this field, as this energy source is not generally considered in equine oocytes.

Other metabolites such as histamine, which has recently been found in equine oviductal fluid [13], are also present in horse FF and could be involved in ovulation induction, as observed in rabbits and rats [39,40] and as also postulated in horses [41].

Our results demonstrate that the base media used for equine IVM and the composition of preovulatory equine FF greatly differ. One limitation of the present study is that the composition of fetal bovine serum (FBS) that is usually added at concentrations ranging from 10% to 20% to equine IVM media has not been considered [4,42]. It is well known that FBS composition greatly varies among batches, and thus, a significant error could be introduced if the composition of a single batch was considered; this is why FBS composition is not considered in the present report.

## 5. Conclusions

In conclusion, our data provide new insights into equine preovulatory follicular fluid composition comparing it with the most widely used commercial media available for equine IVM (TCM-199 and DMEM/F-12). Our data provide new metabolite information that should be considered to design specific equine IVM media and help to improve current in vitro fertilization outcomes in the equine species. More research is warranted to better understand the metabolic requirements of equine oocytes and the relationship among metabolism, oocyte meiotic competence, and developmental competence.

## Figures and Tables

**Table 1:** Chemical shift assignment, multiplicity, and number of contributing protons for the identified metabolites.

Metabolite Name	Chemical Shift (ppm)	Multiplet Type	Protons Contributing
Acetylcarnitine	3.8	dd	
	3.2	s	9
	2.6	dd	
Acetate	1.91	s	3
Alanine	1.46	d	3
	3.76	q	
Aspartate	3.89	dd	2
	2.66	dd	
Carnitine	3.41	m	
	3.21	s	9
	2.42	m	
Choline	3.18	s	9
Citrate	2.52	d	2
Creatine	3.02	s	3
Creatine phosphate	3.03	s	3
Fumarate	6.51	s	2
Glucose	5.22	d	
	3.88	dd	
	3.72	m	1
	3.52	dd	
	3.45	m	
	3.23	dd	
Glucose-1-phosphate	3.75	m	1
	3.39	t	
Glycine	3.54	s	2
Histamine	7.99	s	1
	7.14	s	
Histidine	7.9	d	1
	7.09	d	
Isoleucine	0.99	d	3
Lactate	4.1	q	
	1.3	d	3
Leucine	3.72	m	
	1.70	m	6
	0.94	t	
Pyruvate	2.4	s	3
Succinate	2.39	s	
Threonine	4.24	m	1
	1.31		
Valine	1.02	m	3
	0.97	d	

Multyplet type: s—singlet; d—doublet; t—triplet; dd—doublet of doublets; q—quadruplet; m—multiplet.

**Table 2:** Concentrations of metabolites detected in equine preovulatory follicular fluid.

Metabolite	Follicular Fluid (mM)
Acetlylcarnitine	0.37 ± 0.2 (0.02–1.148)
Acetate	1.5 ± 0.6 (0.5–4.1)
Alanine	1.1 ± 0.14 (0.74–1.6)
Aspartate	2.7 ± 0.3 (2.1–3.7)
Carnitine	0.09 ± 0.01 (0.04–0.14)
Choline	0.03 ± 0.01 (0.01–0.09)
Citrate	0.4 ± 0.04 (0.3–0.5)
Creatine	0.36 ± 0.14 (0.15–0.9)
Creatine phosphate	0.36 ± 0.05 (0.2–0.6)
Fumarate	0.05 ± 0.007 (0.03–0.07)
Glucose	4.3 ± 0.4 (3.1–5.5)
Glucose-1-phosphate	6.9 ± 0.4 (5.75–8.9)
Glycine	3.2 ± 0.5 (1.26–4.8)
Histamine	0.25 ± 0.01 (0.2–0.27)
Histidine	0.05 ± 0.009 (0.04–0.07)
Isoleucine	0.6 ± 0.07 (0.4–0.9)
Lactate	27.3 ± 2.2 (19.3–35.02)
Leucine	0.5 ± 0.05 (0.4–0.8)
Pyruvate + Succinate	0.16 ± 0.03 (0.08–0.3)
Threonine	0.35 ± 0.03 (0.14–0.8)
Valine	0.13 ± 0.02 (0.09–0.2)

The results are presented as mean ± SEM (minimum value−maximum value); the values correspond to 6 different mares (*n* = 6).

**Table 3:** Presence and concentration of the metabolites found in equine preovulatory follicular fluid, and in TCM-199 and DMEM/F-12 media (manufacturer’s specifications).

Metabolite	Follicular Fluid (mM)	TCM-199 (mM)	DMEM/F-12 (mM)
Acetate	1.5	0.61	-
Acetlylcarnitine	0.37	-	-
Alanine	1.1	0.28	0.05
Aspartate	2.7	0.22	0.05
Carnitine	0.09	-	-
Citrate	0.4	-	-
Creatine	0.36	-	-
Creatine phosphate	0.36	-	-
Choline	0.03	0.003	0.064
Fumarate	0.05	-	-
Glucose (d-Glucose)	4.3	5.55	17.5
Glucose-1-phosphate	6.9	-	-
Glycine	3.2	0.67	0.25
Histamine	0.25	-	-
Histidine	0.05	0.1	0.15
Isoleucine	0.6	0.3	0.41
Lactate	27.3	-	-
Leucine	0.5	0.46	0.45
Pyruvate + Succinate	0.16	-	0.5
Threonine	0.35	0.25	0.45
Valine	0.13	0.21	0.45

## Supplementary Materials

The following are available online at https://www.mdpi.com/2076-2615/10/5/883/s1, Figure S1. Metabolite assignment on 1D ^1^H NMR spectra of an FF sample. (**A**) Chemical shift region from 6.4 to 8.0 ppm, (**B**) chemical shift region from 3 to 4.2 ppm, (**C**) chemical shift region from 1.8 to 2.5 ppm, (**D**) chemical shift region from 0.8 to 1.5 ppm.
