# Supplementary material for: Study of the Metabolomics of Equine Preovulatory Follicular Fluid: A Way to Improve Current In Vitro Maturation Media
Source: Animals (Basel). 2020 May 19;10(5):883. doi: 10.3390/ani10050883 (PMC7278476; doi:10.3390/ani10050883)
Supplement: Supplementary file 1 [file animals-10-00883-s001.pdf]

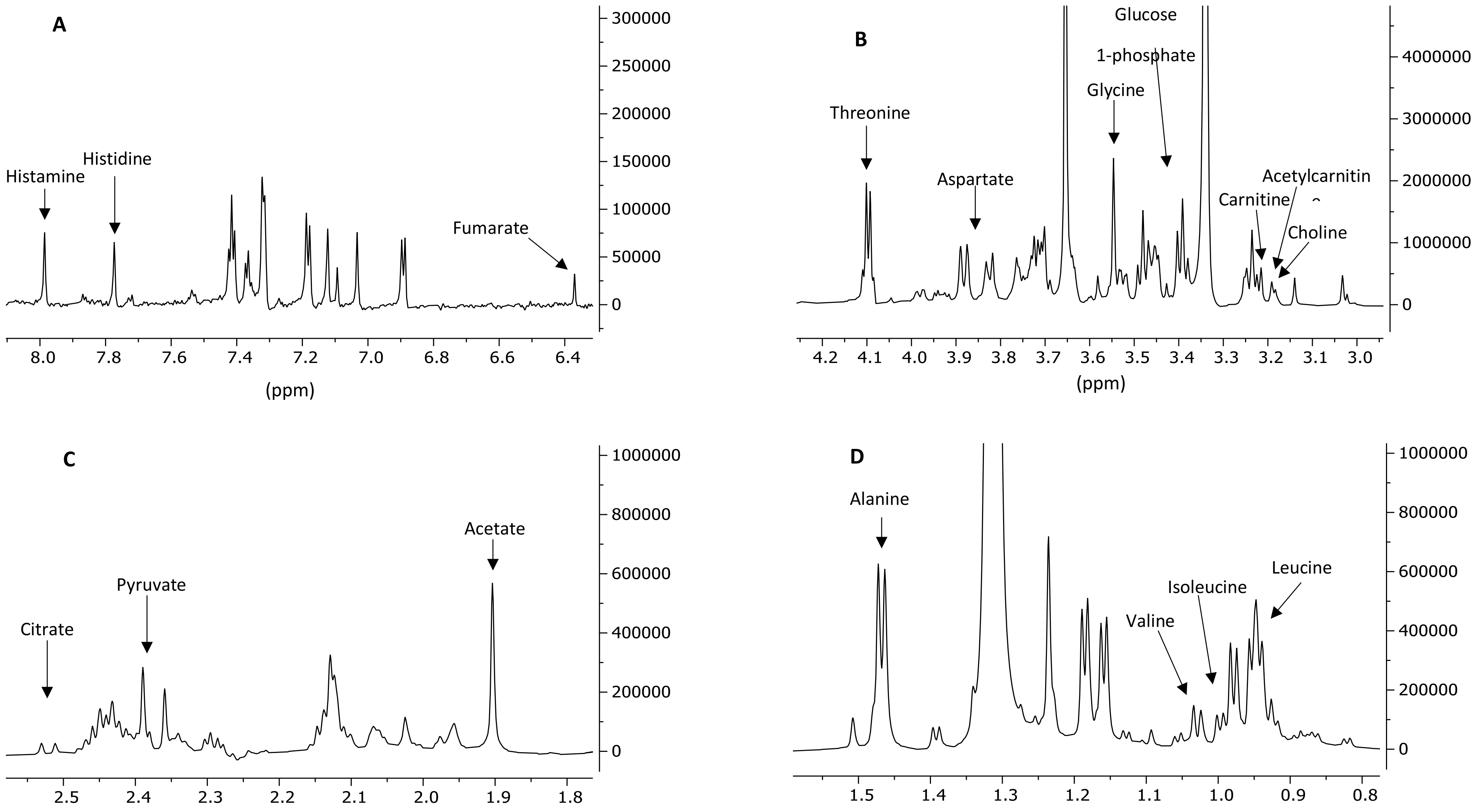

Figure S1. Metabolite assignment on 1D ^{1}H NMR spectra of an FF sample. (A) chemical shift region from 6.4 to 8.0 ppm, (B) chemical shift region from 3 to 4.2 ppm, (C) chemical shift region from 1.8 to 2.5 ppm, (D) chemical shift region from 0.8 to 1.5 ppm.
